# Real-world Prevalence of Nonintegrase INSTI Resistance-Associated Mutations and Virological Outcomes in People Who Have Recently Acquired HIV-1 in the United Kingdom

**DOI:** 10.1093/infdis/jiaf500

**Published:** 2025-09-26

**Authors:** Christine Kelly, James S Lester, Daniel Bradshaw, David F Bibby, Hodan Mohamed, Gary Murphy, Alison Brown, Caroline Sabin, Anna-Maria Geretti, Jean L Mbisa

**Affiliations:** Virus Reference Department, UK Health Security Agency, Colindale, United Kingdom; Centre for Experimental Pathogen Host Research, University College Dublin, Dublin, Ireland; Virus Reference Department, UK Health Security Agency, Colindale, United Kingdom; Blood Safety, Hepatitis, Sexually Transmitted Infections and HIV Division, UK Health Security Agency, London, United Kingdom; Virus Reference Department, UK Health Security Agency, Colindale, United Kingdom; Virus Reference Department, UK Health Security Agency, Colindale, United Kingdom; Virus Reference Department, UK Health Security Agency, Colindale, United Kingdom; Virus Reference Department, UK Health Security Agency, Colindale, United Kingdom; Blood Safety, Hepatitis, Sexually Transmitted Infections and HIV Division, UK Health Security Agency, London, United Kingdom; Institute for Global Health, University College London, London, United Kingdom; National Institute for Health and Care Research Health Protection Research Unit in Bloodborne and Sexually Transmitted Infections, University College London, London, United Kingdom; Department of Medicine of Systems, University of Rome Tor Vergata, Rome, Italy; Department of HIV Medicine, Royal Free London NHS Trust (NorthMid), London, United Kingdom; Virus Reference Department, UK Health Security Agency, Colindale, United Kingdom; Blood Safety, Hepatitis, Sexually Transmitted Infections and HIV Division, UK Health Security Agency, London, United Kingdom; National Institute for Health and Care Research Health Protection Research Unit in Bloodborne and Sexually Transmitted Infections, University College London, London, United Kingdom

**Keywords:** HIV, ART, INSTI, resistance, mutations

## Abstract

**Background:**

Integrase strand transfer inhibitors (INSTIs) are the mainstay of antiretroviral therapy (ART) globally. Virological breakthrough is uncommon but often manifests as low-level viremia, and only 50% of cases have identified drug resistance mutations in the integrase gene. Nonintegrase mutations in the Gag-nucleocapsid protein (NC), envelope glycoprotein (Env), and 3′ polypurine tract (3′PPT) have been identified in vitro.

**Methods:**

Between 2015 and 2021, human immunodeficiency virus type 1 (HIV-1) whole genome sequencing was performed on samples from people with recently acquired HIV-1 in the United Kingdom. Sequences were linked to demographic and clinical data within the UK Health Security Agency's HIV and AIDS Reporting System. The relationship between nonintegrase enzyme mutations and virological outcomes was assessed. Of 1106 participants, 375 (34%) started an INSTI-based regimen. Of these, 337 (90%) were men and 196 (52%) were living with subtype B. The median age was 33 years and number of viral loads within 24 months of starting ART was 4.

**Results:**

Overall, Env Y61H (33 [10%]), A539V (16 [5.0%]), 3′PPT c9053t (17 [5.0%]), and NC N8S (16 [4.8%]) were the most prevalent nonintegrase enzyme mutations. Univariable and multivariable Cox regression did not identify significant associations between the presence of these mutations individually and time to viral suppression, or to viral blip. Interestingly, accessory INSTI mutations were found significantly more frequently in people whose virus also harbored the Env mutation A539V (*P* = .002).

**Conclusions:**

Several nonintegrase mutations were prevalent, but we found no evidence of an impact upon virological outcomes within treatment-naive individuals on INSTI-based regimens who had recently acquired HIV.

## Teaser

Common transmitted nonenzyme integrase strand transfer inhibitor mutations were not found to affect virological outcomes in isolation. The effect of accumulation of multiple such mutations alone or in combination with enzyme mutations should be the focus of future research.

## Graphical abstract

**Graphical abstract:**
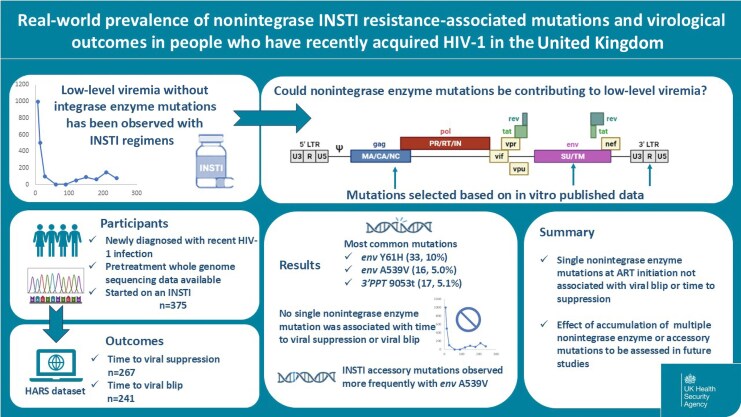

Integrase strand transfer inhibitors (INSTIs) are now the mainstay of first-line treatment for human immunodeficiency virus type 1 (HIV-1) across the world [1]. In the United Kingdom (UK), the British HIV Association guidelines recommend the use of the INSTIs dolutegravir and bictegravir as the additional agent in combination with nucleoside reverse transcriptase inhibitor (NRTI) treatment for initiation of therapy in adults with HIV [2]. Part of the reason for their success is a high genetic barrier to resistance, especially for the second-generation INSTIs that include dolutegravir, bictegravir, and cabotegravir [3]. The latter is part of the only currently approved long-acting injectable regimen (cabotegravir/rilpivirine), which can also be used as a single agent for HIV preexposure prophylaxis. However, INSTI resistance does arise and in studies of regimens containing dolutegravir in low- and middle-income countries, resistance in the integrase gene has been detected in 3.9% to 19.6% of individuals experiencing treatment failure [4]. In addition, INSTI resistance has been associated with nonintegrase mutations in the 3′PPT [5], the envelope glycoprotein (Env) [6], and the Gag-nucleocapsid protein (NC) [7], the clinical consequences of which are poorly defined. Within the viral life cycle, 3′PPT facilitates immune escape and infectivity of HIV-infected cells by modulating the expression of surface proteins [8], while Env is key to binding and fusion with host cells [9] and NC fulfils a number of roles, including facilitating viral assembly, chaperoning viral RNA, and stabilizing viral DNA [10].

Mutations within the 3′PPT region have been implicated in dolutegravir resistance in vitro [5]. These consist of a replacement of the cytidine by a thymine in position 9053, and the modification of the subsequent GGGGGG sequence from position 9069 to 9073 to GCAGT, with a deletion in position 9073. Now unable to integrate, 1-LTR DNA circles are formed which under the correct cellular conditions induce production of select viral proteins [11]. Although these viral products are unlikely to be a source of productive virus, they could contribute to detection of viral blips [12].

Several Env mutations have been implicated in dolutegravir resistance, including Y61H and P81S in gp120, and A539V and A556T in gp41 [6]. Of these, virus with the A539V mutation in particular displays enhanced cell-to-cell transmission, unimpaired cell-free transmission, and significant reduction in sensitivity to dolutegravir and reduced sensitivity to other antiretroviral classes [13]. The other 3 mutations have been associated with the enhancement of cell-to-cell but impairment of cell-free transmission, alongside a reduction in sensitivity to dolutegravir. This enhancement of cell-to-cell transmission, seemingly mediated by the increased stability of Env conformations and decreased gp120 shedding, is thought to be the primary mechanism of dolutegravir resistance in these instances. In vitro, NC mutations, typically alongside Env mutations described above such as A539V, accelerate viral DNA integration leading to an exceptionally high multiplicity of infection, which can overwhelm the antiviral effect of INSTIs [7, 10].

Virological failure on a second-generation INSTI–based treatment typically manifests as low-level viremia and in the absence of drug resistance mutations within the integrase gene [3, 14]. This absence of recognized mutations raises questions as to the potential role for the described nonintegrase mutations. Understanding if and how their detection should play a role in clinical practice is a critical element of future-proofing this antiretroviral therapy (ART) class.

Routinely, HIV drug resistance testing genotypes enzymes specific to antiretroviral drug classes. Whole genome sequencing (WGS) has been provided as a reference surveillance service for a subset of new HIV diagnoses at the UK Health Security Agency (UKHSA) national laboratory and World Health Organization Global Specialised HIV Drug Resistance Laboratory in the UK since 2015. It affords the additional opportunity to examine resistance patterns outside of genotyped regions. Here, we link WGS data with clinical metadata to investigate the relationship between mutations that affect INSTI efficacy and virological outcomes.

## MATERIALS AND METHODS

### Population

The UKHSA has sequenced HIV whole genomes from all individuals identified to have recently acquired HIV-1 in the UK since 2015 as part of routine HIV surveillance. All samples with sufficient leftover volume that have undergone avidity testing for recency of acquisition were eligible for WGS. Recent acquisition, defined as being in the preceding 6 months, is determined by the recent infection testing algorithm (RITA), which uses the results of an HIV-1 antibody avidity assay and clinical characteristics. RITA excludes those with evidence of treatment at time of sampling, and so the sequences to which WGS was applied were strictly pretreatment. RITA results were available for approximately 21% of those newly diagnosed during 2015–2021.

All individuals with available UKHSA WGS data, and linked clinical metadata from the UK’s HIV and AIDS Reporting System (HARS), were eligible for inclusion. For the analysis of mutation prevalence, we excluded individuals whose RITA result did not confirm recent acquisition. For the analysis of time to viral suppression and blip, we then further excluded those who did not start treatment on an INSTI-based regimen, had no viral load (VL) within 12 months of starting treatment, or had a missing or suppressed VL at baseline. For the analysis of time to viral blip, we further excluded those who were no longer on an INSTI-based regimen at time of viral suppression, those who did not have VLs available after viral suppression, and those who did not reach viral suppression within 24 months of starting treatment.

### Laboratory Methods

The presence of clinically relevant drug resistance mutations (surveillance drug resistance mutations [SDRMs]) was identified by submitting sequences to the Stanford Calibrated Population Resistance tool [15]. This tool both identifies established SDRMs and applies sequence and mutation-level exclusion criteria to remove potentially spurious findings based on factors including low coverage, multiple APOBEC mutations, or adjacency to insertions, deletions, or frame shifts [16, 17]. We also identified accessory INSTI resistance mutations using the Stanford HIVdb program to assess the co-occurrence of known integrase mutations with the mutations of interest [18]. Subtypes were obtained using the Stanford HIVdb program and the COMET HIV-1 subtyping tool [18, 19]. Concordance between the 2 methodologies was high (99.6%), with Stanford able to assign a higher portion of samples to a subtype (93.7% vs 66.3%) and thus used for subsequent analysis.

The complete genomic sequencing of HIV was performed using a previously described sequence capture method and the short read data were assembled into consensus whole genomes using Genomancer, an in-house viral genome assembly pipeline [20].

Consensus sequences derived at 30× depth and 20% variant frequency threshold were aligned against the HXB2 reference sequence using MAFFT, and mutations of interest were then identified either within the aligned 3′PPT nucleotide sequence or translated Env or NC amino acid sequence as appropriate [21]. Where reads at a given locus were ambiguous, mutation presence was considered unknown. Throughout this manuscript we have used position numbering consistent with how these mutations were originally described, rather than their actual HXB2 positions, but it should be noted that Env 209, 539, and 556 are instead 211, 541, and 558 in HXB2 and thus our alignments. Similarly, the 3′PPT positions of interest referred to as 9053, 9069, 9070, 9072, and 9073 correspond to 9063, 9079, 9080, 9082, and 9083. NC positions were unchanged.

### Outcomes

Our primary outcome was a viral blip defined as a VL >50 copies/mL after having reached viral suppression. Although the etiology of detectable VLs on ART is complex and multifactorial, the occurrence of viral blips carries an increased risk of future virological failure [22]. Because virological failure is now a rare occurrence, we selected viral blip as a pragmatic virological outcome. For this outcome people were followed from viral suppression while on an INSTI-based treatment, to date of death, change to a non-INSTI-based regimen, or final available VL. A VL threshold of 50 copies/mL was used for these outcome measures to account for INSTI resistance often manifesting as persistent low-level viremia. Data across all available time periods for included people were used.

The secondary outcome was time to viral suppression post–ART initiation, defined as time from the start of treatment to the first VL ≤50 copies/mL. For this outcome, people were followed from ART initiation with an INSTI-based treatment to date of death, change to a non-INSTI-based regimen (including both change to a protease inhibitor [PI]–based regimen or the addition of a PI to the regimen), or final available VL. Follow-up was limited to 24 months after ART initiation.

### Variable Management

The ART start date, baseline VL, subsequent VL, and ART regimen all required derivation from HARS.

### ART Start Date

ART initiation is captured by both explicit ART start dates and ART status at attendances. For the purposes of analysis, ART start date was taken to be the earliest of either a reported ART start date, or the first of 2 consecutive attendances where an individual was reported to be on treatment. If an individual had evidence of viral suppression before this date, treatment initiation was backdated to date of baseline VL collection.

### Baseline VL

Where a VL was available at ART initiation, this was used. If not available, the highest VL within 30 days before or 7 days after was used, otherwise the nearest value within 1 year before starting ART, otherwise the nearest within 1 week after, or finally the nearest value at any time before starting ART.

### Viral Load

Viral load was occasionally ambiguously reported as 0 to indicate either an undetectable VL, or where VL was not tested, and as such was treated as missing data. Where a VL was reported as being between 1 and 5, this was assumed to have been log transformed and was corrected accordingly. Repeated identical VL values >100 copies/mL were assumed to reflect the results of previous tests that had been carried forward, with subsequent duplicated values treated as missing data.

### ART Regimen

When available, an individual's starting regimen was taken to be their first recorded ART regimen within 1 year of ART initiation, provided it contained no ambiguous medications (eg, other unknown, other PI). When ambiguity was present within this first recorded regimen, clarifications within 1 year of starting ART were taken to reflect this first regimen. Where multiple distinct regimens were listed on the same day, the more complete regimen was used. If both were equally complete, they were merged.

### Statistical Analysis

To identify associations between the mutations of interest and individual and virological characteristics, we used the Wilcoxon test for continuous variables, the χ^2^ test for categorical variables where all expected values were ≥5, and Fisher exact test otherwise. We used univariable and multivariable Cox regression to identify any associations between time to viral suppression and viral blip and the mutations of interest. Univariable Cox regression was used to identify variables for inclusion in a multivariable regression, with all variables with a *P* < .1, and key characteristics (age and gender) included in the multivariable regression.

### Ethics

Ethical approval was obtained through the UKHSA Research Ethics and Governance Group on 5 April 2022 (reference number NR0303) and the UKHSA Caldicott Advisory Panel on 29 November 2022 (reference number CAP-2022-18).

## RESULTS

### Population Identification

**Figure 1.**
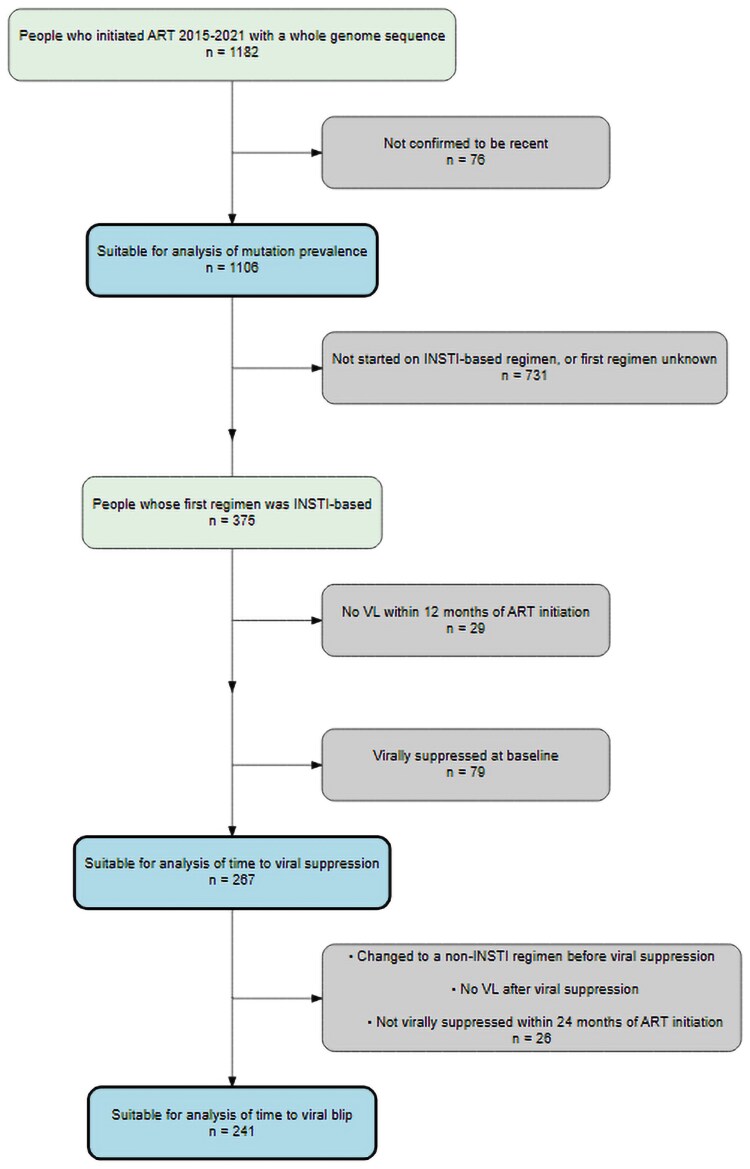
Inclusion criteria flowchart. Abbreviations: ART, antiretroviral therapy; INSTI, integrase strand transfer inhibitor; VL, viral load.

A total of 1182 people had available WGS data between 2015 and 2021. Of these, 76 HIV acquisitions could not be confirmed to be recent (Figure 1).

Sequentially excluding individuals who did not start on an INSTI-based regimen or whose first regimen was unknown (n = 731), those with no VL within 12 months of ART initiation (n = 29), and those who were virally suppressed at baseline (n = 79) left 267 people suitable for analysis of time to viral suppression, our secondary outcome. A further 26 people were excluded from the analysis of time to subsequent viral blip, our primary outcome, due to switching to a non-INSTI-based regimen before reaching viral suppression (n = 10), having no recorded VL after viral suppression (n = 13), or no viral suppression (n = 3) within 24 months of starting treatment. Of those with no viral suppression within 24 months, none had evidence of any of the mutations of interest.

### Demographic and Clinical Characteristics

The overall population comprised 990 (90%) men and 806 (75%) people of White ethnicity (Table 1). The most common probable acquisition route (816 [78%]) was sex between men. The majority had been born in the UK (655 [63%]) and had HIV subtype B (610 [55%]). No important demographic differences were noted between the overall sample and those who were on an INSTI-based regimen. Most people had a baseline CD4 count ≥350 cells/µL (862 [79%]) and an initial HIV VL of ≥100 000 copies/mL (483 [55%]).

**Table 1.** Baseline Characteristics of the Study Population

Characteristic	Started on an INSTI-Based Regimen (n = 375)	Started on Any Regimen (Including Unknown) (n = 1106)
Age at ART start, y, median (Q1, Q3)	33 (26, 41)	32 (26, 42)
Gender		
Men (including transgender men)	337 (90)	990 (90)
Women (including transgender women)	38 (10)	115 (10)
Nonbinary	0 (0)	1 (<0.1)
Ethnic group		
White	269 (74)	806 (75)
Black	35 (9.6)	101 (9.4)
Asian	28 (7.7)	71 (6.6)
Other/Mixed	32 (8.8)	95 (8.9)
Unknown	11	33
Probable route of HIV-1 acquisition		
Sex between men	265 (77)	816 (78)
Sex between men and women	75 (22)	220 (21)
Other	3 (0.9)	10 (1.0)
Unknown	32	60
Region of birth		
United Kingdom	210 (60)	655 (63)
Africa	27 (7.7)	74 (7.1)
Americas	20 (5.7)	60 (5.7)
Asia and Oceania	33 (9.4)	74 (7.1)
Europe	62 (18)	183 (17)
Unknown	23	60
Baseline CD4 count		
<350 cells/µL	73 (20)	227 (21)
≥350 cells/µL	290 (80)	862 (79)
Unknown	12	17
Baseline VL		
<100 000 copies/mL	132 (45)	394 (45)
≥100 000 copies/mL	160 (55)	483 (55)
Unknown	83	229
Viral subtype		
B	196 (52)	610 (55)
C	47 (13)	101 (9.1)
CRF01_AE	29 (7.7)	72 (6.5)
CRF02_AG	34 (9.1)	100 (9.0)
F1	17 (4.5)	53 (4.8)
Other (n < 50)	52 (14)	170 (15)

Data are presented as No. (%) unless otherwise indicated.

Abbreviations: ART, antiretroviral therapy; HIV-1, human immunodeficiency virus type 1; INSTI, integrase strand transfer inhibitor; Q1, quartile 1; Q3, quartile 3; VL, viral load.

### Antiretroviral Therapy

Those who started on an INSTI-based regimen had typically started their treatment more recently than those starting other regimens and had done so on a raltegravir-based (205 [55%]) or dolutegravir-based (137 [37%]) regimen, with smaller portions of the sample receiving elvitegravir (5 [1.3%]) or bictegravir (28 [7.5%]). Thus, 56% started on a first-generation INSTI, and 44% on a second-generation INSTI. Overall, individuals had a median of 5 VLs available within 24 months of ART initiation, and a median duration of follow-up of 19 months. This was consistent across all treatment groups. Further details on major drug resistance mutations can be found in the Supplementary Materials.

### Genotypic Resistance Prevalence

NRTI SDRMs were most prevalent (44 [4.1%]), followed by nonnucleoside reverse transcriptase inhibitor SDRMs (35 [3.2%]) and PI SDRMs (29 [2.7%]). INSTI SDRMs were seen in <1% of sequences (detailed breakdown available in the Supplementary Material).

### Nonintegrase Enzyme INSTI Resistance Mutations

Of the 3′PPT, Env, and NC mutations of interest, 3′PPT c9053t (47 [4.7%]), Env Y61H (102 [11%]), Env A539V (36 [3.9%]), and NC N8S (52 [5.2%]) were most prevalent.

The presence of 3′PPT 9053t was significantly associated with both region of birth and viral subtype (Table 2). Env Y61H was significantly associated with a lower baseline VL and absence of INSTI accessory mutations. Env A539V was significantly associated with age at ART initiation, ethnic group, region of birth, baseline VL, and viral subtype. Individuals carrying a virus with this mutation were typically older (median age, 38 vs 32 years), were more commonly of an ethnicity other than White (43% vs 25%), were born outside the UK (59% vs 36%), were more likely to have a non-B subtype (86% vs 44%), and more often had at least 1 INSTI accessory mutation (22% vs 7.8%). NC N8S was significantly associated with younger age (median age, 30 vs 32 years) and viral subtype B. Two of the 8 participants with a HIV virus displaying INSTI SDRMs also had this mutation.

**Table 2.** Associations Between Nonintegrase Resistance Mutations and Individual and Virological Characteristics

Characteristic	All	3′PPT c9053t		Env Y61H		Env A539V		NC N8S	
	(N = 1106)	Present (n = 47)	*P* Value^a^	Present (n = 102)	*P* Value^a^	Present (n = 36)	*P* Value^a^	Present (n = 52)	*P* Value^a^
Age at ART start, y, median (Q1, Q3)	32 (26, 42)	32 (26, 39)	.16	30 (25, 44)	.41	38 (31, 47)	.**01**	30 (23, 37)	.**02**
Gender			.22		.07		.41		.84
Men (including transgender men)	990 (90)	45 (96)		97 (95)		31 (86)		47 (90)	
Women (including transgender women)	116 (10)	2 (4.3)		5 (4.9)		5 (14)		5 (9.6)	
Ethnic group			.37		.96		.**02**		.38
White	806 (75)	37 (80)		75 (75)		20 (57)		41 (80)	
All other ethnic groups	267 (25)	9 (20)		25 (25)		15 (43)		10 (20)	
Probable route of HIV-1 acquisition			.16		.38		.34		.13
Sex between men	816 (78)	39 (87)		72 (75)		25 (71)		44 (86)	
All other routes of HIV-1 acquisition	230 (22)	6 (13)		24 (25)		10 (29)		7 (14)	
Region of birth			.**03**		.54		.**004**		1.00
United Kingdom	665 (64)	36 (80)		62 (67)		14 (41)		33 (65)	
Born outside the UK	367 (36)	9 (20)		30 (33)		20 (59)		18 (35)	
Baseline CD4			.96		.07		.67		.16
<350 cells/µL	227 (21)	10 (21)		15 (15)		9 (26)		7 (13)	
≥350 cells/µL	862 (79)	37 (79)		87 (85)		26 (74)		45 (87)	
Baseline VL			.26		.**04**		.**04**		.98
<100 000 copies/mL	394 (45)	20 (51)		44 (51)		7 (23)		20 (43)	
≥100 000 copies/mL	483 (55)	19 (49)		42 (49)		23 (77)		27 (57)	
Viral subtype			**<.001**		.25		**<.001**		**<.001**
B	610 (55)	40 (85)		50 (49)		5 (14)		42 (81)	
Non-B	495 (45)	7 (15)		52 (51)		31 (86)		10 (19)	
Any INSTI SDRMs present	8 (0.7)	0 (0)	1.00	1 (1.0)	.29	0 (0)	1.00	2 (3.8)	.**05**
Any PI SDRMs present	29 (2.7)	1 (2.2)	1.00	1 (1.0)	.50	1 (2.8)	1.00	3 (5.9)	.18
Any NRTI SDRMs present	44 (4.1)	1 (2.1)	.72	2 (2.0)	.42	0 (0)	.40	2 (3.8)	1.00
Any NNRTI SDRMs present	35 (3.2)	2 (4.3)	.67	2 (2.0)	.76	0 (0)	.63	0 (0)	.41
Any INSTI accessory mutations present	85 (7.8)	0 (0)	.07	1 (1.0)	.**01**	8 (22)	.**002**	2 (3.8)	.58

Data are presented as No. (%) unless otherwise indicated. Bold: *P* < .05

Abbreviations: 3′PPT, 3′ polypurine tract; ART, antiretroviral therapy; Env, envelope; HIV-1, human immunodeficiency virus type 1; INSTI, integrase strand transfer inhibitor; NC, nucleocapsid; NNRTI, nonnucleoside reverse transcriptase inhibitor; NRTI, nucleoside reverse transcriptase inhibitor; PI, protease inhibitor; Q1, quartile 1; Q3, quartile 3; SDRM, surveillance drug resistance mutation; UK, United Kingdom; VL, viral load.

^a^Wilcoxon rank-sum test; Pearson χ^2^ test; Fisher exact test.

### Multivariable Analysis of Associations Between Nonintegrase Enzyme INSTI Mutations and Virological Outcomes

**Figure 2.**
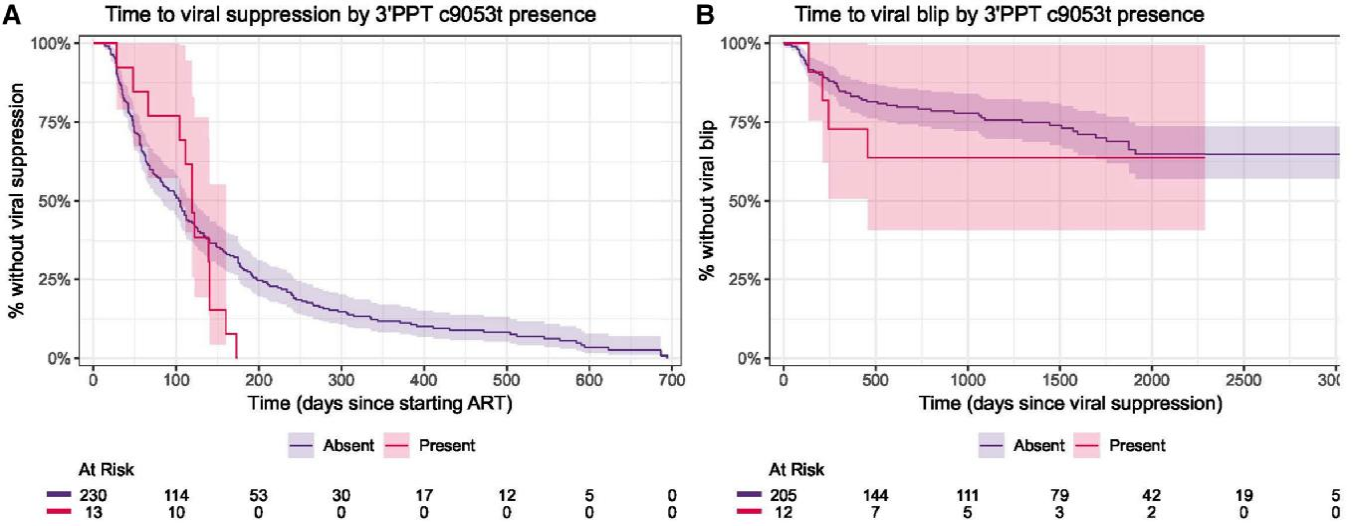
Kaplan-Meier curves of time to viral suppression (*A*) and viral blip (*B*), stratified by the presence of 3′PPT c9053t. Abbreviations: 3′PPT, 3′ polypurine tract; ART, antiretroviral therapy.

**Figure 3.**
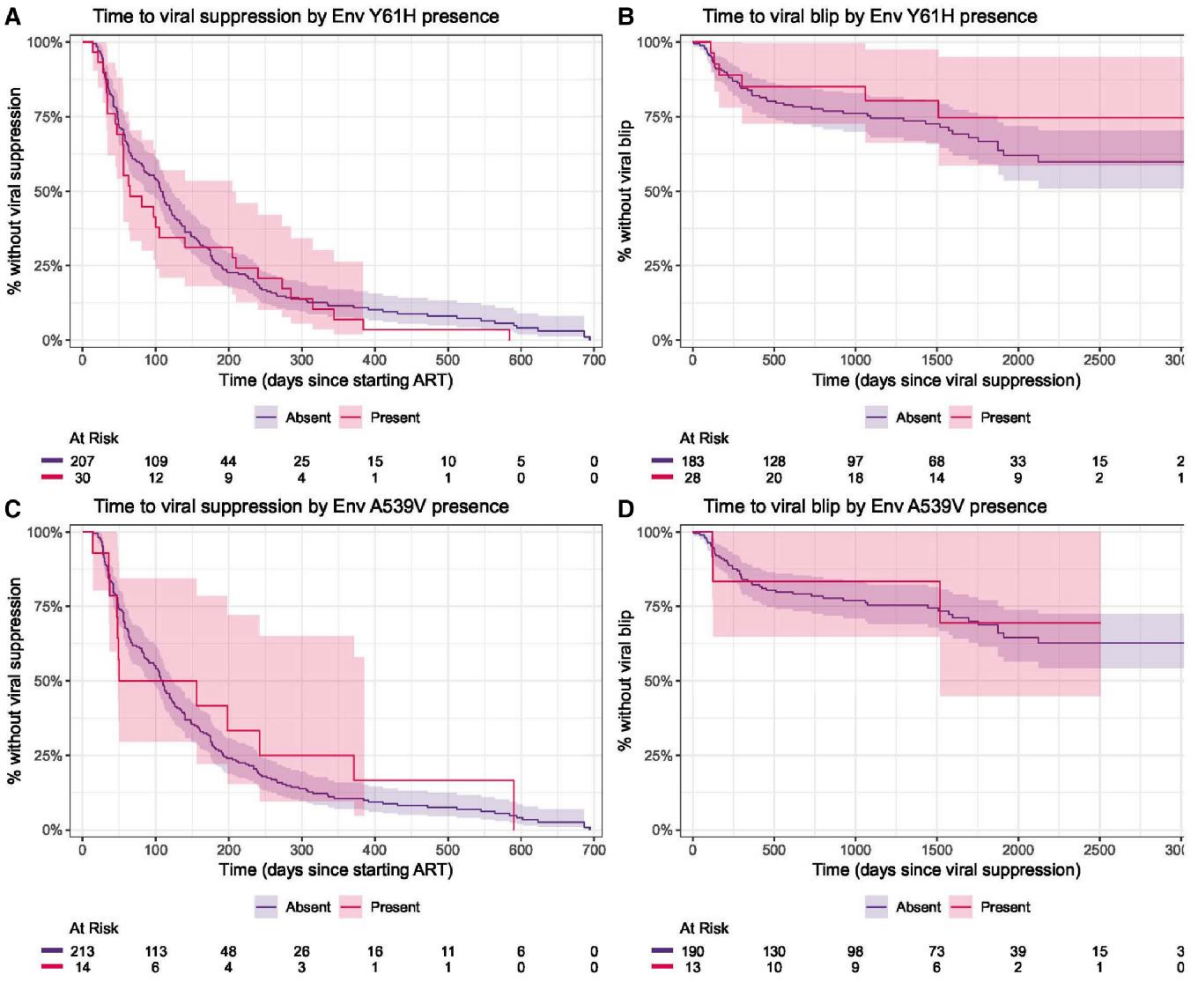
Kaplan-Meier curves of time to viral suppression (*A* and *C*) and viral blip (*B* and *D*), stratified by the presence of Env Y61H (*A* and *B*) and Env A539V (*C* and *D*). Abbreviation: ART, antiretroviral therapy.

**Figure 4.**
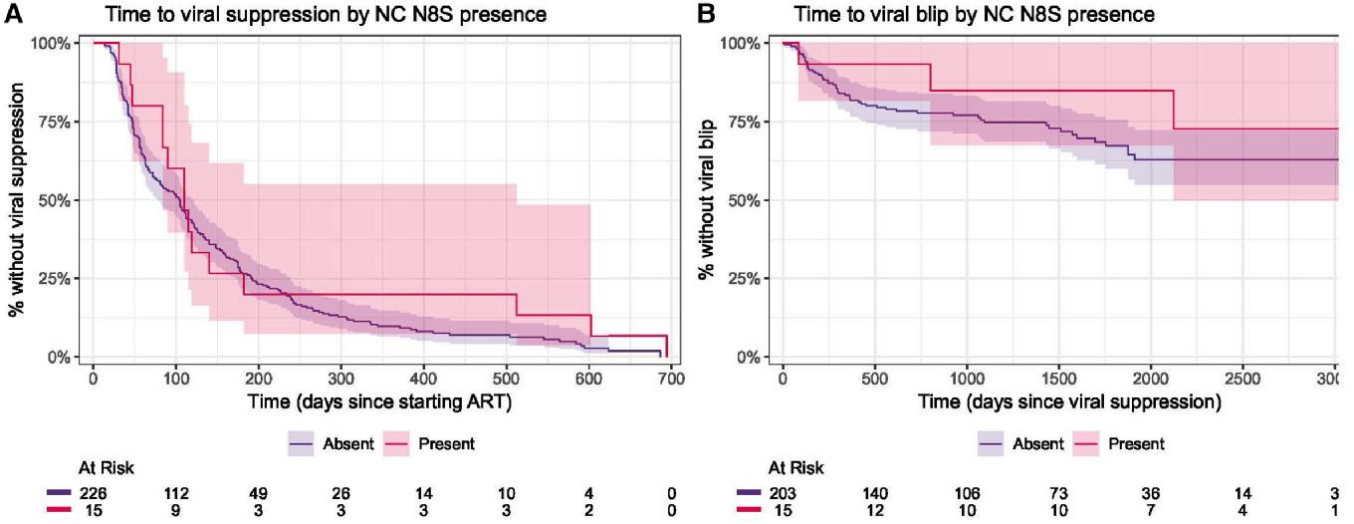
Kaplan-Meier curves of time to viral suppression (*A*) and viral blip (*B*), stratified by the presence of nucleocapsid N8S. Abbreviations: ART, antiretroviral therapy; NC, nucleocapsid.

For the analysis of time to viral suppression, there were 103 person-years of follow-up, and the median time to detecting viral suppression was 89 days. Of 267 individuals who started treatment, 248 had reached viral suppression within 24 months, 8 switched regimens before reaching viral suppression, and 11 did not have evidence of viral suppression before their final VL. For viral blip, a total of 66 events were observed over 815 person-years of follow-up. Univariable Cox analysis identified female gender, lower baseline CD4, and higher baseline VL as associated with a longer time to viral suppression, and female gender as associated with a shorter time to viral blip. No significant effect was observed for any of the nonintegrase mutations under consideration in univariable analysis for time to viral suppression (hazard ratio [95% confidence interval]) (3′PPT c9053t: 1.31 [.74–2.31]; Env Y61H: 1.18 [.79–1.74]; Env A539V: 0.88 [.49–1.58]; NC N8S: 0.73 [.42–1.26]) or for time to viral blip (3′PPT c9053t: 1.44 [.52–3.97]; Env Y61H: 0.66 [.28–1.54]; Env A539V: 0.83 [.26–2.67]; NC N8S: 0.57 [.18–1.82]) (Figures 2–4). These results remained nonsignificant after adjustment for other variables (Table 3). Two subanalyses were performed, 1 limiting analysis of both outcomes only to those on second-generation INSTIs, yielding consistent results, and a second censoring those with possible virological failure after viral blip (VL >50 copies/mL following blip), again yielding consistent results (Supplementary Material).

**Table 3.** Univariable and Multivariable Cox Regression Associations Between Nonintegrase Mutations and Outcomes

Characteristic	Viral Suppression (n = 267)			Viral Blip (n = 241)		
	HR	(95% CI)	*P* Value	HR	(95% CI)	*P* Value
Unadjusted analysis						
3′PPT c9053t						
Present	1.31	(.74–2.31)	.3	1.44	(.52–3.97)	.5
Env Y61H						
Present	1.18	(.79–1.74)	.4	0.66	(.28–1.54)	.3
Env A539V						
Present	0.88	(.49–1.58)	.7	0.83	(.26–2.67)	.8
NC N8S						
Present	0.73	(.42–1.26)	.3	0.57	(.18–1.82)	.3
Adjusted analysis^a,b^						
3′PPT c9053t						
Present	1.29	(.71–2.35)	.4	1.60	(.58–4.46)	.4
Env Y61H						
Present	1.19	(.78–1.81)	.4	0.75	(.32–1.76)	.5
Env A539V						
Present	0.77	(.41–1.42)	.4	0.96	(.30–3.14)	>.9
NC N8S						
Present	0.59	(.34–1.04)	.071	0.59	(.18–1.90)	.4

Abbreviations: 3′PPT, 3′ polypurine tract; CI, confidence interval; Env, envelope; HR, hazard ratio; NC, nucleocapsid.

^a^Analyses of viral suppression adjusted for age, gender, region of birth, baseline CD4 count, and baseline viral load.

^b^Analyses of viral blip adjusted for age, gender, and baseline CD4 count.

## DISCUSSION

We have identified 3′PPT c9053t, Env Y61H, Env A539V, and NC N8S at approximately 5% and higher prevalence within this sample. Three of these mutations displayed significant associations with subtype, with 2 also significantly associated with region of birth in univariable analysis. This is unsurprising given that people born abroad who are diagnosed with HIV in the UK have often acquired their HIV prior to arrival, and thus the subtype of their HIV reflects local circulating subtypes. Marked difference in the prevalence of these mutations by subtype, which may in part reflects natural polymorphisms, are exemplified by A539V where a majority of sequences with this mutation were subtype CRF02_AG. However, analysis of the “Web alignments” from the Los Alamos HIV database using the AnalyzeAlign tool indicated that the A539V mutation is seen in the majority of subtype G sequences, whereas the same is not seen of CRF02_AG itself (Supplementary Material), in contrast to what we observed. This may reflect an aspect of diversity within this subtype that is not currently captured by the sequences included, or overrepresented within our sample.

We identified a low prevalence of transmitted INSTI SDRMs, with a relevant mutation seen in <1% of all suitable sequences, but a higher prevalence of integrase accessory mutations. The presence of A539V was significantly associated with INSTI accessory mutations, which is of interest given the putative role of A539V as a facilitator for the accumulation of INSTI resistance mutations.

We did not see any significant impact of the nonintegrase mutations considered, either in univariable nor multivariable analysis, on time to virological suppression or viral blip. Though these mutations have been shown to play a role directly, or in facilitating INSTI resistance in vitro, this was not replicated in vivo within our sample. Hikichi et al have recently demonstrated through in vitro experiments that A539V was one of the first Env mutations to appear during serial passage under increasing concentrations of dolutegravir, conferring 6-fold resistance to dolutegravir [7]. Accumulation of further Env mutations was necessary to cause high-level resistance with >2000-fold resistance demonstrated in viruses containing 7 mutations. Therefore, a series of Env mutations may be necessary for clinically detectable VL. However, A539V viruses do exhibit a replication advantage compared to wild type and may be an indication of risk of accumulation of further Env mutations in individuals with suboptimal adherence over longer periods of time. This is somewhat substantiated by the coexistence of A539V with known INSTI accessory mutations here, although the numbers were too small to examine any virological impact. Follow-up studies are required to assess the dynamics of Env mutation accumulation for patients with A539V viruses at treatment initiation. Investigation is also required on the extent to which cell-to-cell transfer contributes to fitness advantage in Env mutation viruses, to what extent this process can contribute to a detectable VL, and whether it can drive accumulation of further Env mutations.

HARS is a dataset for national HIV surveillance, and so while it provides nationally comprehensive and detailed longitudinal data, certain details are not requested, or subject to the real-world context of HIV care. As a result, significant heterogeneity exists in characteristics such as exactly when VLs are collected, and valuable predictors such as adherence are not routinely collected. Cleaning of VLs included correcting potentially log-transformed values. We note that our conclusions were unchanged when we experimentally excluded these values entirely. Missingness was present in recorded antiviral regimens, which was handled conservatively. The number of individuals with the mutations of interest was relatively small in this pretreatment population, precluding our ability to disentangle effects of mutation from subtype. Poor virological outcomes were uncommon, reflecting both the efficacy of INSTI-based ART and the high-quality HIV care received, but potentially impairing the statistical power of our analysis. Further, although viral blips are predictive of subsequent virological failure, a minority go on to experience this outcome [22]. Viral blips are multifactorial in etiology and may be driven by factors other than resistance, such as large viral reservoir, infection-driven homeostatic proliferation, and isolated adherence issues [22, 23]. Therefore, viral blips for patients studied here do not necessarily imply drug resistance. However, all of these factors may increase the risk of development of future resistance and virological failure, potentiated by the presence of nonintegrase INSTI resistance mutations in pretreatment viruses.

These data justify further work investigating the role of nonintegrase mutations in the development of INSTI resistance driving virological and clinical failure. Future studies could investigate pretreatment and on-treatment WGS longitudinally, and ideally over a longer time period. This could be coupled with in vitro phenotypic susceptibility testing using replication-competent and subtype-specific recombinant virus systems. Of particular interest may be the mechanisms through which various nonintegrase mutations confer resistance in vivo, and the extent to which viral blips are indicative of the presence of replication-competent virus.

Our results indicate that the presence of individual mutations outside the integrase gene does not have a significant impact on viral blips or time to suppression when present at ART initiation. Relationships between integrase gene and nonintegrase gene mutations are interesting and warrant further study.

## Supplementary Material

jiaf500_Supplementary_Data

## Supplementary Data

Supplementary materials are available at *The Journal of Infectious Diseases* online (http://jid.oxfordjournals.org/). Supplementary materials consist of data provided by the author that are published to benefit the reader. The posted materials are not copyedited. The contents of all supplementary data are the sole responsibility of the authors. Questions or messages regarding errors should be addressed to the author.

## Notes

***Author contributions.*** The analysis was conceived by D. B., A.-M. G., C. K., J. S. L., J. L. M., and C. S. Data were collected by A. B., G. M., and H. M. Data analysis was performed by D. F. B., C. K., and J. S. L. The manuscript was written by C. K. and J. S. L.

***Acknowledgments.*** We gratefully acknowledge the people with HIV, clinicians, microbiologists, immunologists, public health practitioners, occupational health doctors and nurses, and other colleagues who contribute to the public health monitoring of HIV and sexually transmitted infections in the UK. We acknowledge members of the National Institute for Health and Care Research (NIHR) Health Protection Research Unit (HPRU) in Blood Borne and Sexually Transmitted Infections Steering Committee: Professor Caroline Sabin (HPRU Director), Dr John Saunders (UKHSA Lead), Professor Catherine Mercer, Dr Hamish Mohammed, Professor Greta Rait, Dr Ruth Simmons, Professor William Rosenberg, Dr Tamyo Mbisa, Professor Rosalind Raine, Dr Sema Mandal, Dr Rosamund Yu, Dr Samreen Ijaz, Dr Fabiana Lorencatto, Dr Rachel Hunter, Dr Kirsty Foster, and Dr Mamoona Tahir.

***Data availability.*** Sequences for the 1106 individuals included within the analyses presented here are available on request.

***Disclaimer.*** The views expressed are those of the author(s) and not necessarily those of the NIHR, UK Health Security Agency, or the Department of Health and Social Care.

***Financial support.*** Funding for the INITiO study was provided by ViiV Healthcare to A.-M. G. INITiO was an investigator-led study, and funding was administered by InformaPRO S.r.l., Rome, Italy. This study was supported by the National Institute for Health and Care Research Health Protection Research Unit (NIHR HPRU) in Blood Borne and Sexually Transmitted Infections at University College London in partnership with the UK Health Security Agency.
