# Supplementary material for: Real-world Prevalence of Nonintegrase INSTI Resistance-Associated Mutations and Virological Outcomes in People Who Have Recently Acquired HIV-1 in the United Kingdom
Source: J Infect Dis. 2025 Sep 26;233(1):49–58. doi: 10.1093/infdis/jiaf500 (PMC12811859; doi:10.1093/infdis/jiaf500)
Supplement: jiaf500_Supplementary_Data [file jiaf500_supplementary_data.zip › jiaf500_Supplementary_Data.docx]

Supplementary Table 1: Presence of INSTI SDRMs

| **Characteristic** | **N = 1,106** |
| --- | --- |
| INSTI SDRM(s) present, n (%) |  |
| E138K | 2 (0.2%) |
| E92G | 1 (<0.1%) |
| R263K | 2 (0.2%) |
| S230R | 1 (<0.1%) |
| T66I | 1 (<0.1%) |
| Y143H | 1 (<0.1%) |
| None | 1,080 (99%) |
| Unknown | 18 |

Supplementary Table 2: Presence of PI SDRMs

| **Characteristic** | **N = 1,106** |
| --- | --- |
| PI SDRM(s) present, n (%) |  |
| F53L | 1 (<0.1%) |
| G73A | 1 (<0.1%) |
| I47V | 2 (0.2%) |
| I85V | 1 (<0.1%) |
| L90M | 14 (1.3%) |
| M46I | 3 (0.3%) |
| M46I+I54V+L76V | 1 (<0.1%) |
| M46I+L90M | 1 (<0.1%) |
| M46IL | 1 (<0.1%) |
| M46L | 2 (0.2%) |
| N88D | 1 (<0.1%) |
| V82A | 1 (<0.1%) |
| None | 1,040 (97%) |
| Unknown | 37 |

Supplementary Table 3: Presence of NRTI SDRMs

| **Characteristic** | **N = 1,106** |
| --- | --- |
| NRTI SDRM(s) present, n (%) |  |
| D67E | 1 (<0.1%) |
| D67G+T215D | 1 (<0.1%) |
| D67N | 3 (0.3%) |
| F77L | 1 (<0.1%) |
| K219E | 1 (<0.1%) |
| K219N | 7 (0.6%) |
| K219Q | 1 (<0.1%) |
| K70R | 1 (<0.1%) |
| L210W | 1 (<0.1%) |
| L74V | 1 (<0.1%) |
| M184I | 2 (0.2%) |
| M184V | 6 (0.6%) |
| M41L | 4 (0.4%) |
| M41L+L210W+T215S | 1 (<0.1%) |
| M41L+L74I+L210W+T215S | 1 (<0.1%) |
| M41L+T215D | 1 (<0.1%) |
| M41L+T215S | 1 (<0.1%) |
| T215D | 4 (0.4%) |
| T215E | 2 (0.2%) |
| T215I | 1 (<0.1%) |
| T215S | 3 (0.3%) |
| None | 1,042 (96%) |
| Unknown | 20 |

Supplementary Table 4: Presence of NNRTI SDRMs

| **Characteristic** | **N = 1,106** |
| --- | --- |
| NNRTI SDRM(s) present, n (%) |  |
| K101E | 3 (0.3%) |
| K101P+K103S | 1 (<0.1%) |
| K103N | 19 (1.7%) |
| K103N+P225H | 3 (0.3%) |
| K103N+Y181C | 1 (<0.1%) |
| K103S | 2 (0.2%) |
| V106M | 1 (<0.1%) |
| Y181C | 2 (0.2%) |
| Y188H | 1 (<0.1%) |
| Y188L | 2 (0.2%) |
| None | 1,051 (97%) |
| Unknown | 20 |

Supplementary Table 5: Anti-retroviral treatment and drug resistance mutation details

| **Characteristic** | **Overall** N = 1,106 | **Started on INSTI-based regimen** N = 375 | **Started on PI-based regimen** N = 201 | **Started on NNRTI-based regimen** N = 100 | **Started on other regimen** N = 176*^1^* | **Started on unknown regimen** N = 254 |
| --- | --- | --- | --- | --- | --- | --- |
| Treatment and followup | | | | | | |
| **Year ART was started, n (%)** |  |  |  |  |  |  |
| 2015 | 188 (17%) | 37 (9.9%) | 41 (20%) | 34 (34%) | 38 (22%) | 38 (15%) |
| 2016 | 132 (12%) | 28 (7.5%) | 30 (15%) | 16 (16%) | 21 (12%) | 37 (15%) |
| 2017 | 202 (18%) | 57 (15%) | 41 (20%) | 22 (22%) | 44 (25%) | 38 (15%) |
| 2018 | 200 (18%) | 77 (21%) | 43 (21%) | 13 (13%) | 25 (14%) | 42 (17%) |
| 2019 | 204 (18%) | 94 (25%) | 31 (15%) | 7 (7.0%) | 30 (17%) | 42 (17%) |
| 2020 | 103 (9.3%) | 48 (13%) | 11 (5.5%) | 5 (5.0%) | 12 (6.8%) | 27 (11%) |
| 2021 | 77 (7.0%) | 34 (9.1%) | 4 (2.0%) | 3 (3.0%) | 6 (3.4%) | 30 (12%) |
| **INSTI in first regimen, n (%)** |  |  |  |  |  |  |
| raltegravir | 225 (56%) | 205 (55%) | NA | NA | 20 (83%) | NA |
| bictegravir | 28 (7.0%) | 28 (7.5%) | NA | NA | 0 (0%) | NA |
| dolutegravir | 140 (35%) | 137 (37%) | NA | NA | 3 (13%) | NA |
| elvitegravir | 6 (1.5%) | 5 (1.3%) | NA | NA | 1 (4.2%) | NA |
| **Number of VLs available within 24 months of starting treatment, Median (Q1, Q3)** | 5 (3, 6) | 4 (3, 6) | 5 (3, 7) | 5 (4, 7) | 5 (3, 7) | 4 (3, 6) |
| **Duration of followup within 24 months of starting treatment (months), Median (Q1, Q3)** | 19 (15, 22) | 19 (16, 22) | 20 (15, 22) | 20 (15, 23) | 19 (13, 22) | 19 (15, 22) |
| Surveillance drug resistance mutations | | | | | | |
| **Any INSTI SDRMs present, n (%)** | 8 (0.7%) | 3 (0.8%) | 1 (0.5%) | 1 (1.0%) | 2 (1.2%) | 1 (0.4%) |
| **Any PI SDRMs present, n (%)** | 29 (2.7%) | 9 (2.5%) | 7 (3.6%) | 4 (4.2%) | 1 (0.6%) | 8 (3.3%) |
| **Any NRTI SDRMs present, n (%)** | 44 (4.1%) | 13 (3.5%) | 8 (4.1%) | 2 (2.0%) | 12 (7.0%) | 9 (3.6%) |
| **Any NNRTI SDRMs present, n (%)** | 35 (3.2%) | 13 (3.5%) | 9 (4.6%) | 1 (1.0%) | 4 (2.3%) | 8 (3.2%) |
| Non-integrase 3'PPT mutations | | | | | | |
| **3'PPT c9053t present, n (%)** | 47 (4.6%) | 17 (5.0%) | 9 (4.9%) | 6 (6.6%) | 6 (3.7%) | 9 (3.8%) |
| **3'PPT g9069c present, n (%)** | 1 (0.1%) | 0 (0%) | 0 (0%) | 0 (0%) | 0 (0%) | 1 (0.5%) |
| **3'PPT g9070a present, n (%)** | 0 (0%) | 0 (0%) | 0 (0%) | 0 (0%) | 0 (0%) | 0 (0%) |
| **3'PPT g9072t present, n (%)** | 0 (0%) | 0 (0%) | 0 (0%) | 0 (0%) | 0 (0%) | 0 (0%) |
| **3'PPT g9073del present, n (%)** | 28 (2.9%) | 8 (2.4%) | 4 (2.3%) | 3 (3.4%) | 3 (1.9%) | 10 (4.5%) |
| Non-integrase Env mutations | | | | | | |
| **Env Y61H present, n (%)** | 102 (11%) | 33 (10%) | 23 (14%) | 6 (7.8%) | 16 (11%) | 24 (12%) |
| **Env P81S present, n (%)** | 0 (0%) | 0 (0%) | 0 (0%) | 0 (0%) | 0 (0%) | 0 (0%) |
| **Env A539V present, n (%)** | 36 (3.9%) | 16 (5.0%) | 3 (1.8%) | 1 (1.3%) | 4 (2.6%) | 12 (5.8%) |
| **Env A556T present, n (%)** | 2 (0.2%) | 0 (0%) | 2 (1.2%) | 0 (0%) | 0 (0%) | 0 (0%) |
| Non-integrase NC mutations | | | | | | |
| **NC N8S present, n (%)** | 52 (5.2%) | 16 (4.8%) | 10 (5.6%) | 2 (2.2%) | 12 (7.5%) | 12 (5.2%) |
| **NC N17S present, n (%)** | 0 (0%) | 0 (0%) | 0 (0%) | 0 (0%) | 0 (0%) | 0 (0%) |
| **NC G19S present, n (%)** | 0 (0%) | 0 (0%) | 0 (0%) | 0 (0%) | 0 (0%) | 0 (0%) |
| **NC G22E present, n (%)** | 0 (0%) | 0 (0%) | 0 (0%) | 0 (0%) | 0 (0%) | 0 (0%) |
| **NC A25V present, n (%)** | 0 (0%) | 0 (0%) | 0 (0%) | 0 (0%) | 0 (0%) | 0 (0%) |
| **NC N27I/K present, n (%)** | 0 (0%) | 0 (0%) | 0 (0%) | 0 (0%) | 0 (0%) | 0 (0%) |
| **NC R29G/M present, n (%)** | 0 (0%) | 0 (0%) | 0 (0%) | 0 (0%) | 0 (0%) | 0 (0%) |
| **NC R32G present, n (%)** | 0 (0%) | 0 (0%) | 0 (0%) | 0 (0%) | 0 (0%) | 0 (0%) |
| **NC R35D/N present, n (%)** | 1 (<0.1%) | 0 (0%) | 0 (0%) | 0 (0%) | 1 (0.6%) | 0 (0%) |
| **NC G40E present, n (%)** | 0 (0%) | 0 (0%) | 0 (0%) | 0 (0%) | 0 (0%) | 0 (0%) |
| **NC G43E present, n (%)** | 0 (0%) | 0 (0%) | 0 (0%) | 0 (0%) | 0 (0%) | 0 (0%) |
| **NC M46I present, n (%)** | 3 (0.3%) | 0 (0%) | 2 (1.1%) | 1 (1.1%) | 0 (0%) | 0 (0%) |
| *^1^*Regimen combining INSTI, PI or NNRTI, or containing only NRTIs (typically due to missingness) | | | | | | |

Supplementary Table 6: Univariable and multivariable Cox regression associations between non-integrase mutations and outcomes (second generation INSTIs only)

|  | Viral suppression (n = 109) | | | Viral blip (n = 100) | | |
| --- | --- | --- | --- | --- | --- | --- |
| **Characteristic** | **HR** | **95% CI** | **p-value** | **HR** | **95% CI** | **p-value** |
| Unadjusted analysis | | | | | | |
| 3'PPT 9053t (unadjusted) |  |  |  |  |  |  |
| Present | 1.18 | 0.47, 2.93 | 0.7 | 0.00 | 0.00, Inf | >0.9 |
| Env Y61H (unadjusted) |  |  |  |  |  |  |
| Present | 1.37 | 0.76, 2.48 | 0.3 | 0.28 | 0.04, 2.14 | 0.2 |
| Env A539V (unadjusted) |  |  |  |  |  |  |
| Present | 1.21 | 0.48, 3.03 | 0.7 | 0.00 | 0.00, Inf | >0.9 |
| NC N8S (unadjusted) |  |  |  |  |  |  |
| Present | 0.70 | 0.22, 2.21 | 0.5 | 0.00 | 0.00, Inf | >0.9 |
| Adjusted analysis*^1,2^* | | | | | | |
| 3'PPT 9053t (adjusted) |  |  |  |  |  |  |
| Present | 1.25 | 0.44, 3.57 | 0.7 | 0.00 | 0.00, Inf | >0.9 |
| Env Y61H (adjusted) |  |  |  |  |  |  |
| Present | 1.50 | 0.78, 2.89 | 0.2 | 0.31 | 0.04, 2.36 | 0.3 |
| Env A539V (adjusted) |  |  |  |  |  |  |
| Present | 1.10 | 0.42, 2.86 | 0.8 | 0.00 | 0.00, Inf | >0.9 |
| NC N8S (adjusted) |  |  |  |  |  |  |
| Present | 0.57 | 0.17, 1.98 | 0.4 | 0.00 | 0.00, Inf | >0.9 |
| *^1^*Analyses of viral suppression adjusted for age, gender, baseline CD4 and baseline VL. | | | | | | |
| *^2^*Analyses of viral blip adjusted for age, gender, and baseline CD4. | | | | | | |
| Abbreviations: CI = Confidence Interval, HR = Hazard Ratio | | | | | | |

Supplementary Table 7: Univariable and multivariable Cox regression associations between non-integrase mutations and viral blip (censoring those with possible virological failure, VL>50 following blip)

|  | Viral blip (n = 241) | | |
| --- | --- | --- | --- |
| **Characteristic** | **HR** | **95% CI** | **p-value** |
| Unadjusted analysis | | | |
| 3'PPT 9053t (unadjusted) |  |  |  |
| Present | 1.39 | 0.43, 4.50 | 0.6 |
| Env Y61H (unadjusted) |  |  |  |
| Present | 0.58 | 0.21, 1.61 | 0.3 |
| Env A539V (unadjusted) |  |  |  |
| Present | 1.19 | 0.37, 3.85 | 0.8 |
| NC N8S (unadjusted) |  |  |  |
| Present | 0.57 | 0.18, 1.82 | 0.3 |
| Adjusted analysis*^1^* | | | |
| 3'PPT 9053t (adjusted) |  |  |  |
| Present | 1.53 | 0.47, 4.99 | 0.5 |
| Env Y61H (adjusted) |  |  |  |
| Present | 0.62 | 0.22, 1.76 | 0.4 |
| Env A539V (adjusted) |  |  |  |
| Present | 1.28 | 0.39, 4.24 | 0.7 |
| NC N8S (adjusted) |  |  |  |
| Present | 0.59 | 0.18, 1.90 | 0.4 |
| *^1^*Analyses of viral blip adjusted for age, gender, and baseline CD4. | | | |
| Abbreviations: CI = Confidence Interval, HR = Hazard Ratio | | | |
