# Supplementary figures and images for: Real-world Prevalence of Nonintegrase INSTI Resistance-Associated Mutations and Virological Outcomes in People Who Have Recently Acquired HIV-1 in the United Kingdom
Source: J Infect Dis. 2025 Sep 26;233(1):49–58. doi: 10.1093/infdis/jiaf500 (PMC12811859; doi:10.1093/infdis/jiaf500)

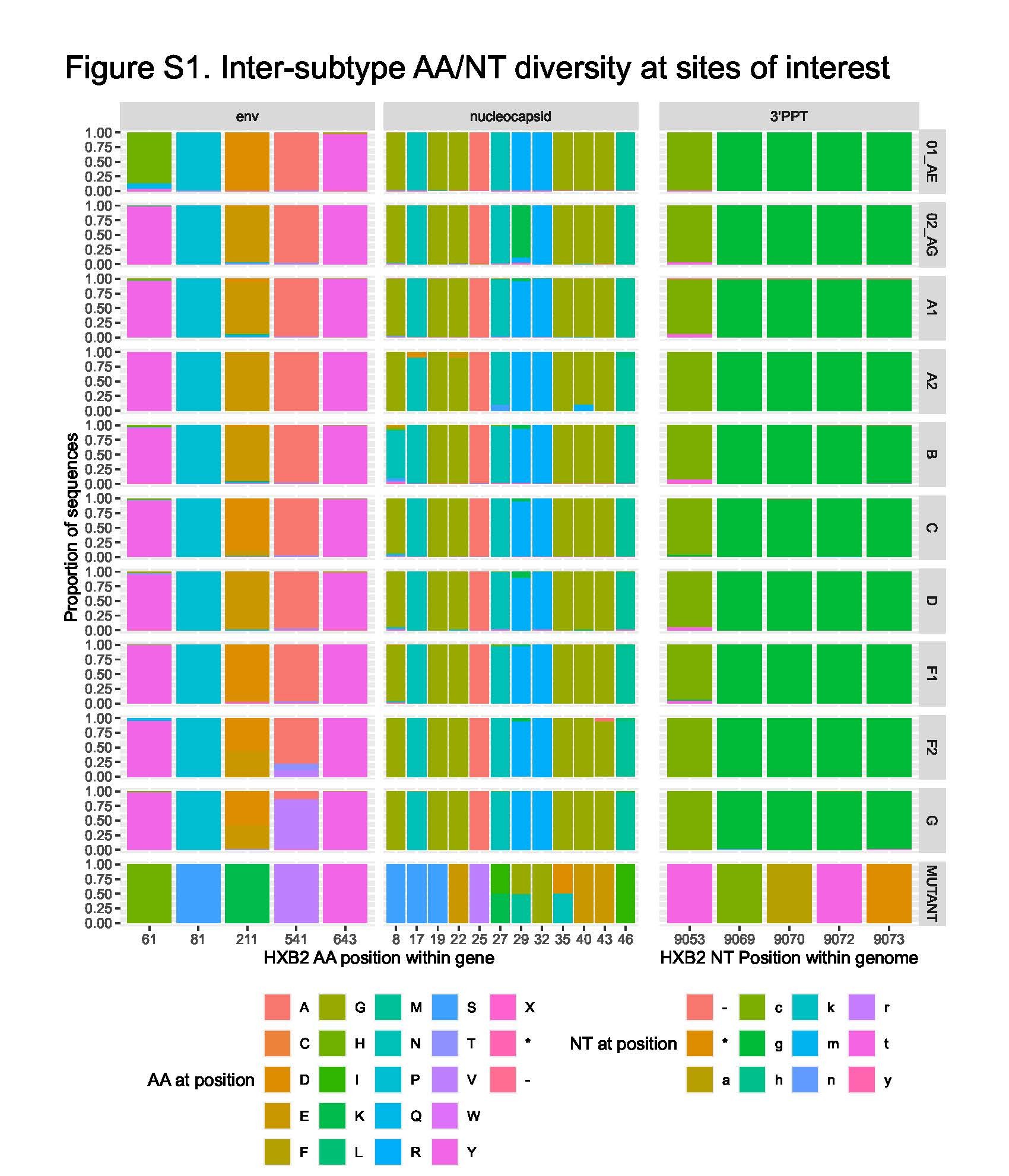

Supplement: jiaf500_Supplementary_Data [file jiaf500_supplementary_data.zip › figure_s1_mutprev.jpg]
